# Combining the high‐dose/refuge strategy and self‐limiting transgenic insects in resistance management—A test in experimental mesocosms

**DOI:** 10.1111/eva.12573

**Published:** 2018-01-18

**Authors:** Liqin Zhou, Nina Alphey, Adam S. Walker, Laura M. Travers, Fevziye Hasan, Neil I. Morrison, Michael B. Bonsall, Ben Raymond

**Affiliations:** ^1^ Imperial College London London UK; ^2^ University of Exeter Exeter UK; ^3^ Department of Zoology University of Oxford Oxford UK; ^4^ The Pirbright Institute Surrey UK; ^5^ Oxitec Ltd Abingdon UK

**Keywords:** Cry1Ac toxin, fitness costs, high‐dose/refuge strategy, resistance management, self‐limiting insects

## Abstract

The high‐dose/refuge strategy has been the primary approach for resistance management in transgenic crops engineered with *Bacillus thuringiensis* toxins. However, there are continuing pressures from growers to reduce the size of Bt toxin‐free refugia, which typically suffer higher damage from pests. One complementary approach is to release male transgenic insects with a female‐specific self‐limiting gene. This technology can reduce population sizes and slow the evolution of resistance by introgressing susceptible genes through males. Theory predicts that it could be used to facilitate smaller refugia or reverse the evolution of resistance. In this study, we used experimental evolution with caged insect populations to investigate the compatibility of the self‐limiting system and the high‐dose/refuge strategy in mitigating the evolution of resistance in diamondback moth, *Plutella xylostella*. The benefits of the self‐limiting system were clearer at smaller refuge size, particularly when refugia were inadequate to prevent the evolution of resistance. We found that transgenic males in caged mesocosms could suppress population size and delay resistance development with 10% refugia and 4%–15% initial resistance allele frequency. Fitness costs in hemizygous transgenic insects are particularly important for introgressing susceptible alleles into target populations. Fitness costs of the self‐limiting gene in this study (*P. xylostella* OX4139 line L) were incompletely dominant, and reduced fecundity and male mating competitiveness. The experimental evolution approach used here illustrates some of the benefits and pitfalls of combining mass release of self‐limiting insects and the high‐dose/refuge strategy, but does indicate that they can be complementary.

## 1 INTRODUCTION

The damage caused by invertebrate pests accounts for 10%–15% of agricultural production, costing approximately US$8 billion in the United States (Metcalf, 1996), US$17.7 billion in Brazil (Oliveira, Auad, Mendes, & Frizzas, 2014), and US$359.8 million in Australia (Murray, Clarke, & Ronning, 2013). One approach to control pests and maintain sustainable agricultural yields is through the use of biopesticides such as *Bacillus thuringiensis (Bt)*. *Bt* is extremely valuable in modern agriculture. This utility results from the insecticidal crystal (Cry) proteins that have high specificity to particular insect groups and hence low toxicity to nontarget organisms (Schnepf et al., 1998). The application of these insecticidal proteins through conventional spray formulations and in transgenic crops can provide effective pest management while maintaining agro‐ecosystem biodiversity (Bravo, Likitvivatanavong, Gill, & Soberon, 2011). Nineteen crops and over 60 million hectares of land have been cultivated with biotech crops expressing *Bt* toxins (James, 2014). However, despite the success of genetically modified (GM) crops, a range of pest species have developed increased levels of resistance to *Bt* biopesticides and to the Cry toxins expressed in GM crops (Gassmann, Petzold‐Maxwell, Keweshan, & Dunbar, 2011; Kruger, Van Rensburg, & Van den Berg, 2011; Storer, Kubiszak, Ed King, Thompson, & Santos, 2012; Tabashnik, Gassmann, Crowder, & Carrière, 2008; Tabashnik, Van Rensburg, & Carrière, 2009; Zhang et al., 2011, 2012). While current resistance management strategies have been effective in a range of species (Carrière, Crowder, & Tabashnik, 2010), there is still scope for improvement and development.

The cornerstone of resistance management for GM crops is the high‐dose/refuge strategy, an approach mandated in several countries. In the high‐dose/refuge strategy, one part of target pest population is exposed to high concentrations (high doses) of toxins produced by *Bt* crops, rendering resistance functionally recessive. When the inheritance of resistance is recessive, only homozygous‐resistant individuals (RR genotype) survive on *Bt* crops. Another proportion of the pest population is maintained in nearby refuges of non‐*Bt* host plants, providing a reservoir of susceptible alleles (from RS and SS genotypes). If the resistance allele frequency is low, homozygous‐resistant pests surviving on *Bt* crops will be relatively rare, while susceptible pests will be abundant and readily available to mate with resistant individuals. Progeny from such matings will be heterozygous for resistance alleles and phenotypically susceptible to high‐dose *Bt* crops, thereby hindering the evolution of resistance. Theoretical models and empirical observations have shown that the high‐dose/refuge strategy is an effective approach to delay or prevent the development of resistance when the above conditions are met (Alphey, Coleman, Bonsall, & Alphey, 2008; Alstad & Andow, 1995; Caprio, Faver, & Hankins, 2004; Gould, 1998; Gryspeirt & Gregoire, 2012; Huang, Andow, & Buschman, 2011; Hutchison et al., 2010; Tyutyunov, Zhadanovskaya, Bourguet, & Arditi, 2008).

The high‐dose/refuge strategy cannot be applied without regard to its basic assumptions. Certain genetic and ecological conditions need to hold true before it can be used to delay the evolution of resistance. These include the following: low initial resistance allele frequency; effectively recessive resistance; and efficient dispersal to refugia. The latter condition includes both random mating between the resistant and susceptible genotypes as well as random oviposition on *Bt* crop and in refugia (Burd, Gould, Bradley, Van Duyn, & Moar, 2003; Frutos, Rang, & Royer, 2008; Liu et al., 2001; Tellez‐Rodriguez et al., 2014). Theoretical models and practical experience have shown that violation of these assumptions of the high‐dose/refuge strategy can lead to rapid evolution of resistance (Alstad & Andow, 1995; Campagne et al., 2016; Caprio et al., 2004; Georghiou & Taylor, 1977; Gould, 1998; Gryspeirt & Gregoire, 2012; Hutchison et al., 2010; Tyutyunov et al., 2008). In addition, if growers fail to plant refugia, then evolution of resistance to GM crops can also be rapid (Farias et al., 2014; Kruger et al., 2011; Monnerat et al., 2015; Storer et al., 2010). Thus, recent incidences of the evolution of resistance to *Bt* toxins in GM crops can largely be traced to failure of the basic assumptions, that is low doses or nonrecessive resistance (Gassmann et al., 2011; Storer et al., 2012) or to the fact that farmers are not adhering to the mandatory refuge planting requirements (Tabashnik, Brevault, & Carrière, 2013).

The high‐dose/refuge strategy can be made more resilient through a range of approaches. These include the use of multiple toxins (“pyramiding”), which further reduces the frequency of effective phenotypic resistance (Carrière, Crickmore, & Tabashnik, 2015; Zhao et al., 2005), and through seed mixes or “refuge in a bag” approaches that enforce farmer compliance (Yang et al., 2014) or manipulation of the fitness costs of resistance using natural enemies or alternative plant varieties (Gassmann, Stock, Sisterson, Carrière, & Tabashnik, 2008; Raymond, Sayyed, Hails, & Wright, 2007; Raymond, Wright, & Bonsall, 2011). Alternative approaches may include the use of transgenic insects to mitigate resistance and to reduce pest population size directly.

Here, we will address experimentally whether the release of transgenic insects to suppress insect population size is compatible with the high‐dose/refuge strategy and can improve its resilience. Recent advances in genetic engineering have enabled the development of transgenic insects carrying a repressible female‐specific lethal gene (Thomas, Donnelly, Wood, & Alphey, 2000). In a strategy mimicking sterile insect technique programmes, the release of large numbers of transgenic males can reduce target populations, as there will be no viable offspring arising from mating of wild females and transgenic males (Alphey, Bonsall, & Alphey, 2009; Alphey, Coleman, Donnelly, & Alphey, 2007; Gentile, Rund, & Madey, 2015; Thomas et al., 2000). As these transgenes are designed to reduce insect fitness and will decline in frequency postrelease, this transgenic approach has been termed “self‐limiting” (Gould, Huang, Legros, & Lloyd, 2008). In addition to suppressing pest population sizes, the mass release of self‐limiting transgenic males can affect the genetic make‐up of pest populations if lethality is targeted only at females, that is female‐specific self‐limiting transgenes. For example, alleles conferring susceptibility to insecticides carried by the transgenic population can be introgressed into the target population through the male line. Deterministic models of the mass release of self‐limiting males show that this technology can be a valuable tool in slowing the evolution of resistance (Alphey et al., 2007, 2009).

Given the importance of the high‐dose/refuge strategy for managing the evolution of resistance in modern agriculture, a significant advance would be to understand how best to combine refugia with the use of transgenic insects bearing female‐specific self‐limiting genes. Theoretically, the mass release of the self‐limiting males could facilitate the planting of smaller refugia while still preventing the evolution of resistance (Alphey et al., 2007, 2009). With increasing release ratios of the self‐limiting insects, the mass release of the genetically engineered males could even reverse resistance development (Alphey et al., 2007, 2009). Smaller refuge sizes may be particularly attractive to farmers who are reluctant to tolerate large refugia or where it is difficult to enforce compliance. The mass release of the self‐limiting males could also potentially help tackle issues like nonrandom mating between resistant and susceptible individuals as a result of different development times and population structure (Cerda & Wright, 2004; Liu, Tabashnik, Dennehy, Patin, & Bartlett, 1999). Local mass release of the self‐limiting insects might also, for example, eradicate resistant populations before they become widespread.

Building on previous work on the high‐dose/refuge strategy and the self‐limiting insects, we will investigate the interaction between the release of self‐limiting transgenic insects and the high‐dose/refuge strategy in mitigating the evolution of resistance in model experimental system using the diamondback moth (DBM), *Plutella xylostella*. DBM is a well‐known and widespread pest of cruciferous crops. Globally, it imposes management costs of US$1.3 billion–US2.3 billion and causes yield losses estimated at US$2.7 billion per annum worldwide (Furlong, Wright, & Dosdall, 2013; Zalucki et al., 2012). Control failure of DBM is a major concern in agriculture, as this species has developed resistance to almost every insecticide applied in the field as well as resistance to microbial *Bt* sprays (Sarfraz & Keddie, 2005; Tabashnik, 1994). Diamondback moth is also a well‐established model for evaluating novel resistance management strategies (Raymond et al., 2007; Zhao et al., 2005). Genetic markers for resistance to the *Bt* toxin Cry1Ac in our resistant line have been well established (Baxter et al., 2011) and this protein can be incorporated into artificial diet at doses that render resistance functionally recessive. Transgenic strains of DBM with female‐specific self‐limiting constructs have been developed (Jin et al., 2013). Evidence of population suppression by the DBM self‐limiting system has been observed in caged continuous generation studies and low numbers of released self‐limiting males have been shown to slow the evolution of resistance to *Bt* in transgenic crucifers (Harvey‐Samuel et al., 2015).

Using DBM populations with known frequencies of Cry1Ac‐resistance alleles, we tested the compatibility of self‐limiting DBM releases with the high‐dose/refuge strategy in single‐generation and multi‐generation experiments. We investigated whether the release of Cry‐susceptible self‐limiting insects could slow or reverse the evolution of resistance at a range of refuge sizes, release ratios, and initial frequencies of resistance. To compare experimental results to previous theoretical and experimental work, we also characterized the fitness costs associated with transgenic constructs and resistance alleles in our experimental set‐up.

## 2 MATERIALS AND METHODS

### 2.1 Experimental conditions and insect populations

All insect populations were reared at 25°C (±1°C) and 45% (±5%) relative humidity, with a 12:12 light/dark cycle. The rearing procedure of DBM followed published protocols (Martins et al., 2012). The construction of the self‐limiting DBM (OX4319L, Oxitec Ltd) has also been described (Jin et al., 2013). In brief, the self‐limiting system has also been implemented in our *Bt*‐susceptible line using sequences from the self‐limiting gene derived from the *doublesex* (*dsx*) gene of pink bollworm (Jin et al., 2013). Sex‐alternate splicing of this *dsx* sequence allows the development of a female‐specific lethal genetic system that is repressible by provision of tetracycline, or suitable analogues, in the larval feed (Jin et al., 2013). The OX4319L moths are denoted as genotype LL, where “L” represents the OX4319L construct insertion (Jin et al., 2013), and are all homozygous‐susceptible to Cry1Ac toxin (genotype SS).

Exogenous *B. thuringiensis* Cry1Ac was purified from *Escherichia coli* JM109 cells carrying the plasmid pGem1Ac, a gift of Dr Neil Crickmore (University of Sussex), following published protocols (Cornforth, Matthews, Brown, & Raymond, 2015). The purified Cry1Ac toxin was incorporated into artificial diet (F9221B, Frontier Agricultural Sciences) to make toxin diet, at doses (0.5 μg/ml) sufficient to cause near‐recessive resistance (Supporting Information: toxin bioassays). Our resistant population, designated VB‐R, was constructed from a Cry1Ac‐resistant population NO‐QAGE (Baxter et al., 2005; Heckel, Gahan, Liu, & Tabashnik, 1999) and a susceptible population Vero Beach, which is the genetic background of the self‐limiting population (VB, Oxitec Ltd). The VB‐R population was constructed by backcrossing a hybrid population of VB and NO‐QAGE into VB, and selecting for resistance to Cry1Ac for three generations. To create a Cry1Ac‐susceptible population with a similar genetic background, we reared VB‐R without toxin selection for five generations (before resistance became fixed); thereafter, we genotyped mated pairs of males and females using the length polymorphism marker for Cry1Ac resistance (Baxter et al., 2011). Our susceptible population VB‐S was then established using 20 pairs of homozygous‐susceptible individuals. PCR conditions for genotyping homozygous‐susceptible alleles were 5 min at 95°C, 30 × (30 s at 94°C, 30 s at 63°C, 1 min at 72°C), 10 min at 72°C, using primers abcc2F (5′‐GGACGTGATCCCGGTGGGCAGCG‐3′) and abcc2R (5′‐CGTGCGGCAGCTTAGTGTAC‐3′). Both the VB‐R and VB‐S populations were nontransgenic (ww genotype, where “w” represents wild type or absence of the “L” construct).

Single and multiple generations, with the same basic design, investigated the impact of transgenic male release on the evolution of resistance to *Bt* toxins (Table 1, details below). Homozygous‐susceptible LL male pupae were introduced into resistant populations with confirmed resistance allele frequencies. Following LL male releases, resistant populations were exposed to toxin selection and refuge treatment. Population size (number of pupae) and resistant frequencies were monitored throughout the experiments.

**Table 1:** Overview of experimental designs

	Release ratio (transgenic males to wild‐type males)	Initial resistance allele frequency	Refuge size	Experiment time
Single‐generation experiment	2:1 & no release	15%	10% & 20%	One discrete generation (2 weeks)
Three‐generation experiments	6:1 & no release	15% & 4%	10%	12 weeks

### 2.2 Single‐generation experiment

These experiments assessed the effect of the susceptible self‐limiting DBM in resistance management at a range of refuge sizes. We hypothesized that the use of susceptible self‐limiting DBM will have a greater effect on slowing the evolution of resistance at smaller refuge sizes. The single‐generation experiments were timed so that wild‐type adults and transgenic males would emerge from their pupae over the same period (24–48 hr) and compete for mates in experimental cages. The eggs produced within each replicate cage were allocated to Cry1Ac toxin diet or toxin refugia where larvae experienced selection for resistance. These experiments sought to control for any differences in development time between wild‐type and transgenic insects (and between Cry1Ac‐resistant and Cry1Ac‐susceptible insects) but otherwise allowed genetic background to affect mating behaviour.

Experiments were set up with 200 individuals of the wild‐type population with a 15% resistance allele frequency (R). The population was reared for at least two generations prior to selection starting and frequencies were confirmed with PCR, using methods described above. In the transgenic LL male release treatment, 200 LL male pupae were added to each replicate, so that the release ratio was 2:1 OX4319L males to wild‐type nontransgenic males. Here, we crossed a refuge size treatment (10% and 20% Cry1Ac toxin‐free refugia) with a transgenic treatment (with and without LL male release), each replicated three times (Table 1). Refugia were based on the percentage of egg population: refugia eggs were reared separately on toxin‐free diet, while remaining eggs were reared on toxin diet (0.5 μg/ml) to pupation. For every replicate, pupae survivors from both the selection diet and refuge diet were collected and pooled for bioassays in the following generation (*N *= 90 larvae and three Cry1Ac doses including 0.131, 0.262 and 0.524 μg/ml) to assess for differences in resistance to Cry1Ac.

### 2.3 Three‐generation experiments

To investigate the value of the self‐limiting DBM in resistance management over multiple generations, we designed two multi‐generation selection experiments with weekly releases of LL males (Table 1). Populations with 4% and 15% resistance allele initial frequencies were generated as above. After confirming the resistant frequency with PCR, we started the first experiment (15% resistance allele frequency) with two treatments (with and without LL male release) and four replicates (400 pupae) in each treatment. In the release treatment, male pupae were introduced into the experimental populations twice a week at approximately a 6:1 ratio (LL male to pupal survivors from each cage, assuming 1:1 sex ratio in cage survivors) for 12 weeks.

Eggs were collected every two days with 10% of the eggs placed onto toxin‐free refuge diet. The diet infestation was staggered every two days to build gradually a continuous population with overlapping generations. Thus, genotype differences in development time or mating success are allowed to influence results, adding more realism than in single‐generation experiments.

The experimental populations were bio‐assayed every generation to measure the proportion of homozygous‐resistant (RR) individuals in the population. Survival data—the numbers of pupae surviving the selection diet and refuge diet—were collected weekly. To test whether the release of transgenic insects was capable of reversion, that is, decreasing the resistance allele frequency in the face of selection, the experiment was repeated with another population with initial resistance allele frequency at 4%.

### 2.4 Life history and fitness cost experiments

To evaluate the fitness costs of the self‐limiting gene and the resistance allele, we measured life history traits and mating competitiveness of the aforementioned *P. xylostella* populations. All males denoted as LL and Lw were homozygous‐susceptible at the resistance locus (SS), and all VB‐S and VB‐R individuals were nontransgenic (ww). We confirmed that the VB‐R population used in this experiment was fixed for resistance by PCR screening of 96 individuals. Single‐pair mating of LL male × SS female, VB‐S individuals (SS), VB‐R individuals (RR) and SS × RR genotype was set up to measure fecundity, egg hatch rate and larval survival until pupation. Single pairs were mated in 106 pots. The number of eggs laid on cabbage juice‐infused green cloths (3 cm × 3 cm) from the single pairs was counted manually for all pots, and eggs were allowed to hatch in situ (Raymond et al., 2007). Twenty freshly emerged neonates from each mating pot were randomly selected to grow on artificial diet until pupation. After scoring survival, pupae developed from single‐pair pots were used in mate competition experiments. In these experiments 10 nontransgenic SS males competed with the same number of LL males, RR males, or hemizygous‐susceptible OX4319L males (LwSS) for mating with 10 SS females. LL males were also competed with hemizygous Lw males for mating with SS females. As the self‐limiting gene contains a dominant heritable, fluorescent DsRed2 protein marker (Jin et al., 2013), pupae can be sorted using a binocular microscope with Nightsea™ light source (excitation 510–540 nm) and 600‐nm filter. Mating success of either LL or Lw males in competition with SS males was scored based on the proportion of fluorescent male offspring. The mating success of RR males was calculated from the proportion of heterozygous‐resistant progeny (RS) using PCR genotyping described above. For Lw males in competition with LL males, the proportion of nonfluorescent male offspring determined the mating success of Lw males.

### 2.5 Statistical analyses and experimental design

To assess the potential discriminatory power of the experiments, we simulated discrete generations of DBM classified by sex and genotype (at L/w and S/R loci), assuming a constant proportion of released LLSS males to emerging males (initial males or, after the first generation, emerging males of any genotype) and random mating. Where known, parameter values were set to match experimental protocols. These simulations were adapted from a previously published discrete‐generation deterministic model of this genetic system in a generic pest insect (Alphey et al., 2007, 2009; see Supporting Information for details). Deterministic model results indicated that the single‐generation experiments were expected to be insensitive to error in allocation of eggs to either toxin or refuge diet. Deterministic modelling showed that the ability to discriminate between treatments over one or three generations is inferior if resistance is more effective and/or if fitness costs of resistance are small. These results informed and refined the experimental design.

Statistical analysis was carried out in R (http://www.r-project.com) using analysis of variance and generalized linear modelling. The numbers of pupal survivors from selection diet and refuge diet in the single‐generation experiment were analysed with a generalized linear model with Poisson errors. Survival data were analysed using a generalized linear mixed model (GLMM) with Poisson errors (Venables & Ripley, 2002); proportional data were analysed with GLMMs with binomial errors, and mixed‐model analyses used replicate as a random effect and nested generation, week and bioassay dose within replicate. Mating success was analysed with a chi‐squared goodness‐of‐fit tests, which compared the expected frequency of L and R alleles under random mating with observed frequencies. All model assumptions were checked with graphical analysis of error distribution assumptions.

## 3 RESULTS

### 3.1 Single‐generation experiment

**Figure 1:**
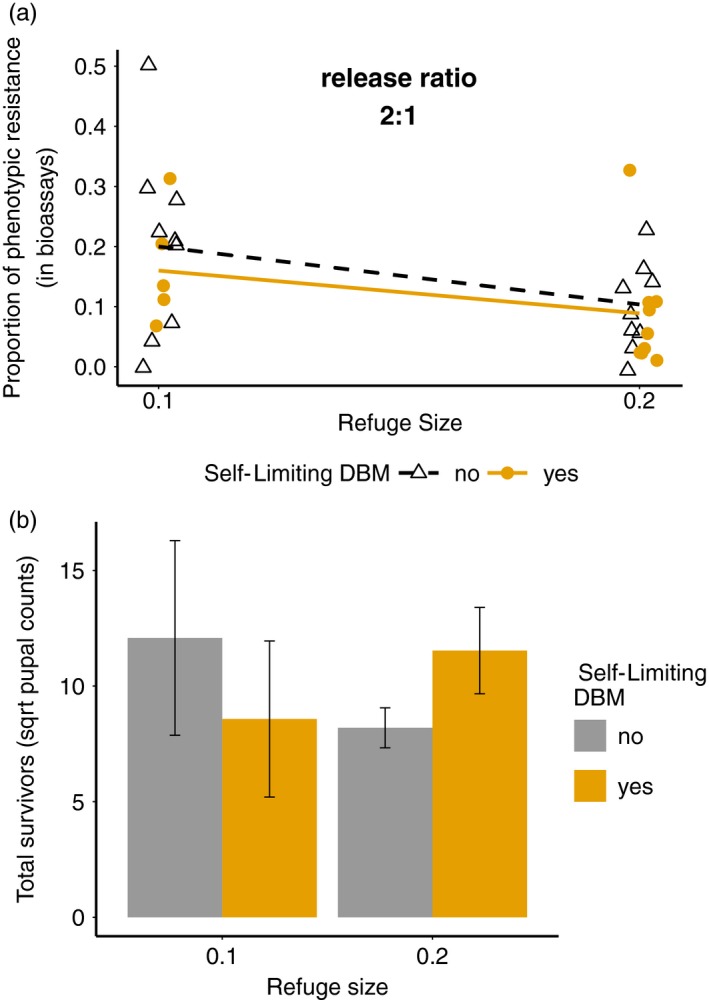
Efficacy of release of transgenic self‐limiting insects in preventing evolution of resistance to *Bt* toxin in single‐generation experiments. (a) Proportion of phenotypic resistance (in bioassays) of populations treated with no release (black open triangles, black dashed line) and release of the self‐limiting DBM males (yellow solid circles, yellow solid line) at 10% and 20% refuge size. (b) Mean total survivors (±*SE*) of populations treated with no release (grey bar) and release of the self‐limiting DBM males (yellow bar) at 10% and 20% refuge size

We predicted that larger refuge sizes and the addition of transgenic males would slow the evolution of resistance. However, given the increased population size associated with larger refugia, we anticipated that the release of transgenic insects would have more impact at smaller refuge sizes. After one discrete generation, at 10% refuge size, one replicate in the release treatment had only five pupal survivors. The replicate went extinct in the following generation and was excluded from bioassays, but was included in the population size analysis. As predicted, the larger refuge size (20%) led to a lower frequency of phenotypic resistance, that is, frequency of RR genotype inferred from bioassay results, compared to replicates with 10% refuge size (Figure 1a, likelihood ratio test = 10.04, *p* = .0015). At 10% refuge size, the addition of transgenic males also lowered the proportion of phenotypic resistance compared to replicates without LL male release treatment (Figure 1a, likelihood ratio test = 8.10, *p* = .0044). However, at 20% refuge size, there was no significant difference between the release and nonrelease treatments (Figure 1a, likelihood ratio test = 0.34, *p* = .56).

The release of transgenic males was also expected to suppress population size by killing female progeny (Alphey et al., 2007, 2009). We define total survivors as the number of surviving pupae pooled from Cry1Ac‐containing diet and refuge diet across replicates. Given an initial R allele frequency of 15%, after one discrete generation, neither refuge size (*F*_1,10_ = 0.025, *p* = .88) nor the release of transgenic males (*F*_1,9_ = 0.0008, *p* = .98) had an impact on the total survivors (Figure 1b).

The single‐generation design is less realistic and has less power than the multiple generation experiment below. In addition to controlling for differences in development time, self‐limiting alleles cannot build up over time in the targeted populations. However, these experiments were informative in terms of illustrating the parameter values (resistance frequency, refuge size, release ratios) over which we might see effects of transgenic insects on evolution of resistance.

### 3.2 Three‐generation experiments

**Figure 2:**
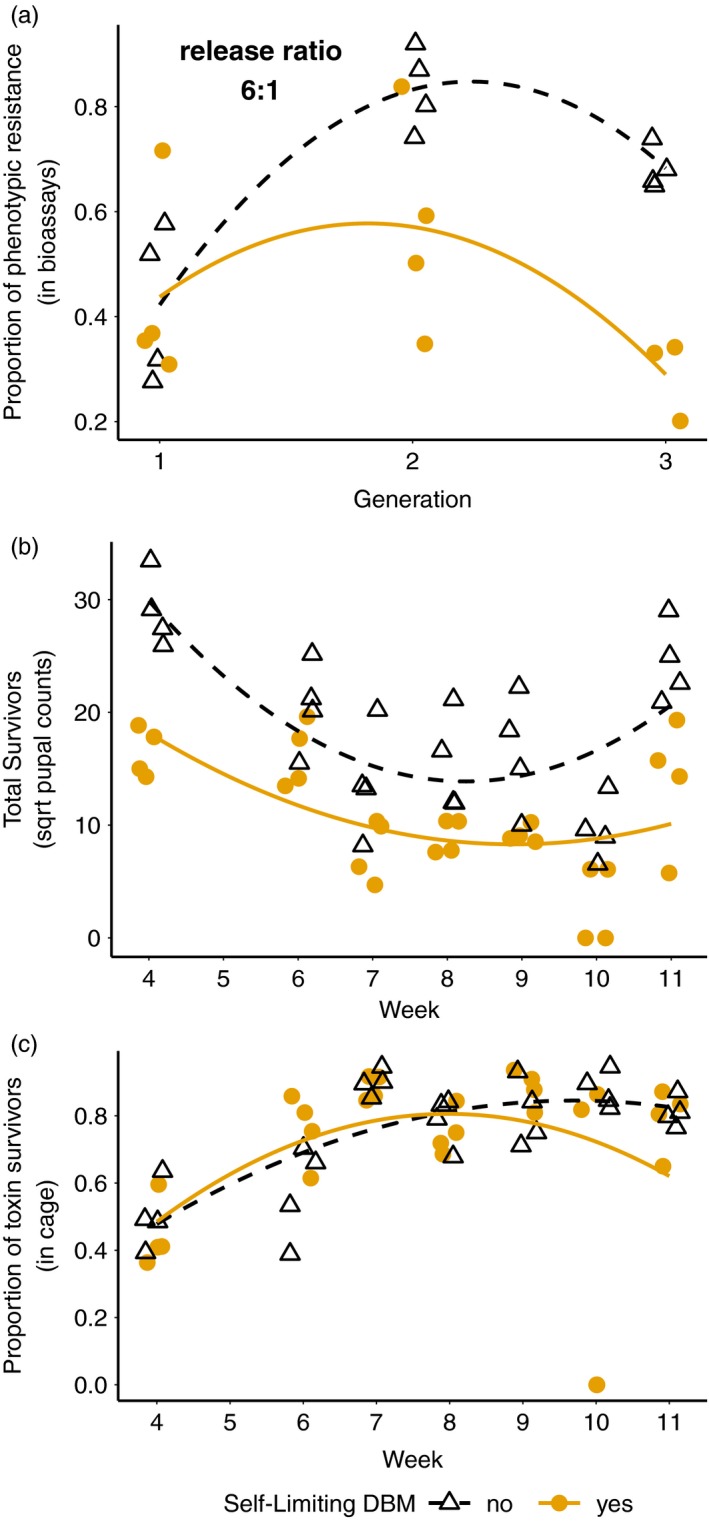
Efficacy of release of transgenic self‐limiting insects in preventing evolution of resistance to *Bt* toxin with continuous generations experiments and high (15%) initial resistance allele frequency. (a) Proportion of phenotypic resistance (in bioassays) of populations treated without LL male release (black open triangles, black dashed line) and with weekly LL male release (yellow solid circles, yellow solid line) over three generations. (b) Total survivors and (c) Proportion of toxin survivors (in cage) of populations treated with nonrelease (black open triangles, black dashed line) and with weekly LL male release (yellow solid circles, yellow solid line) over 7 weeks’ time points. Proportion of toxin survivors represent the ratio of homozygous‐resistant survivors (RR pupae) from Cry1Ac selection diet to total pupae survivors pooled from selection diet and refuge diet in each cage population. Experiments used a 10% refuge size

While the single‐generation experiment showed an effect on resistance frequency at lower refuge size, it was weaker than that predicted by theory. Here, we hypothesized that a higher release ratio of transgenic males in a continuous generation experiment should produce a more robust impact on both population size and resistance frequency as transgene frequencies are expected to increase over time in target populations under continuous release. In the first multi‐generation experiment, initial conditions were the following: initial resistance allele frequency of 15%, and a 10% refuge size, and a release ratio of 6:1 transgenic: wild‐type males. Under these conditions, the release of transgenic males significantly reduced phenotypic resistance compared to controls without release (Figure 2a, treatment * generation interaction, likelihood ratio test = 11.94, *p* < .001; treatment * generation^2^ interaction, likelihood ratio test = 3.99, *p* = .046). Model comparison showed that a GLMM model with quadratic interaction between treatment and week (AIC = 359.07) had greater explanatory power than a model with a linear interaction (AIC = 442.74; chi‐squared test = 89.67, *df *= 3*, p* <<< .001).

In addition to phenotypic resistance reduction, we also observed population size suppression (Figure 2b, treatment * week interaction, likelihood ratio test = 6.28, *p* = .012; treatment * week^2^ interaction, likelihood ratio test = 6.19, *p* = .013). Model comparison showed that the effects of time were nonlinear; adding week as a quadratic term improved model fitting (treatment * week^2^ interaction, AIC = 355.09, chi‐squared test = 42.61, *df* = 3, *p* <<< .001) relative to a simple linear analysis (AIC = 385.62). Note that larval populations on toxins and refugia diets crashed in week 10, due to undiagnosed issues in the insectary; populations rebounded in week 11 as adults and eggs in each replicate were unaffected by this additional mortality. We also estimated the selective advantage of resistance in experiments from the proportion of insects that survived on Cry1Ac‐containing diet relative to total pupal survivors. If there is no effective resistance, then this value should be 0; while if resistance is at fixation, this value should be equal to (1‐ refuge size), or 0.9 with a 10% refuge. Over the course of the experiment, the proportion of Cry1Ac survivors increased in both released populations and controls (Figure 2c, likelihood ratio test = 4.06, *p* = .044). We also observed an increase in Cry1Ac survivors at first and later (after week 10) a decrease in Cry1Ac survivors in populations treated with transgenic males (Figure 2c, treatment * week interaction, likelihood ratio test = 33.49, *p* <<< .001; treatment * week^2^ interaction, likelihood ratio test = 31.85, *p* <<< .001). A quadratic interaction between treatment and week (AIC = 645.24) had greater explanatory power than a linear interaction (AIC = 678.39; chi‐square test = 37.15, *df* = 2, *p* <<< .001).

Following the success of the first experiment, we tested whether we could drive reversion (decrease in frequency) of resistance in populations initiated with 4% R allele frequency. In this experiment, population sizes across all treatments and replicates (5~158 pupae) were lower than in the experiment with 15% initial R allele frequency (115~1,516 pupae), a consequence of the lower mean reproductive ability associated with reduced phenotypic resistance. Particularly after two generations, all four replicates with weekly release of LL males had zero survivors from Cry1Ac diet and a very low number of survivors from refuge diet (5~72 pupae). Nevertheless, we found support for reduction in population sizes and frequency of resistance alleles in treatments with release of transgenic males.

**Figure 3:**
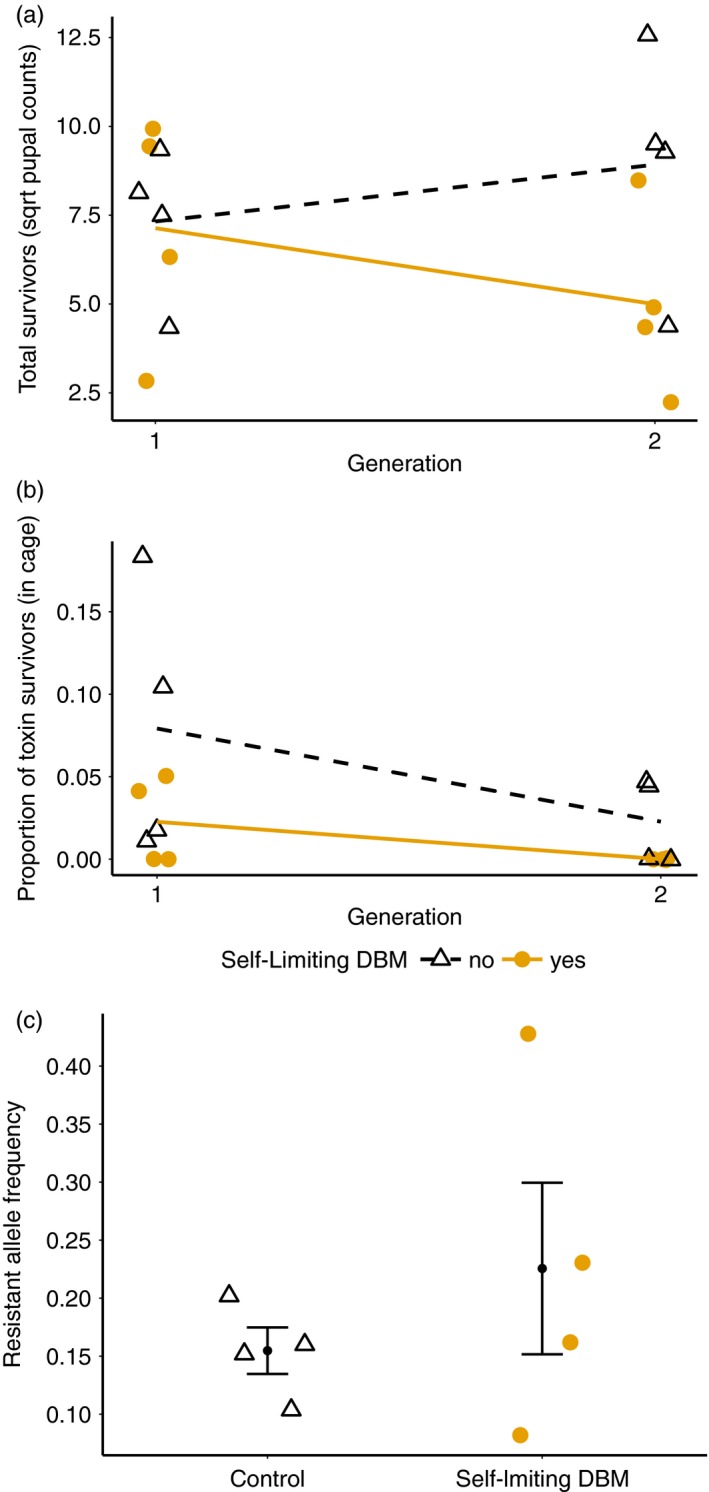
Efficacy of release of transgenic self‐limiting insects in preventing evolution of resistance to *Bt* toxin with continuous generations experiments and low (4%). (a) Total survivors and (b) Proportion of toxin survivors (in cage) of populations treated with nonrelease (black open triangles, black dashed line) and with weekly LL male release (yellow solid circles, yellow solid line) over two generations. Proportion of observed resistant represent the ratio of homozygous‐resistant survivors (RR pupae) from Cry1Ac selection diet to total pupae survivors pooled from selection diet and refuge diet in each population. (c) Resistance allele frequency of populations treated without LL male release (black open triangles) and with weekly LL male release (yellow solid circles) at the second generation. Black solid circles and error bars represent the mean resistance allele frequency (±*SE*) for respective treatments. Experiments used a 10% refuge size

The release of transgenic males reduced population size by generation 2 (Figure 3a, treatment * generation interaction, likelihood ratio test = 65.51, *p* <<< .001). The release of transgenic males also significantly reduced the proportion of the population surviving on Cry1Ac over the course of experiment (Figure 3b, likelihood ratio test = 10.66, *p* = .0011). Notably, by generation 2, no insects survived on Cry1Ac diet in the transgenic release treatment. After the second generation of LL male release, population replicates did not produce enough third‐instar larvae for bioassays. As a result, R allele frequency was confirmed by PCR instead of bioassay, and the experiment was terminated at the second generation. Despite the effect of the release of LL males on the Cry1Ac survivors, transgenic insects did not significantly affect the frequency of R alleles after selection (Figure 3c, *F*_1,6_ = 0.85, *p* = .39), quite possibly because genetic drift/bottleneck effects in refugia confounded experimental treatments.

### 3.3 Life history and fitness cost experiments

**Figure 4:**
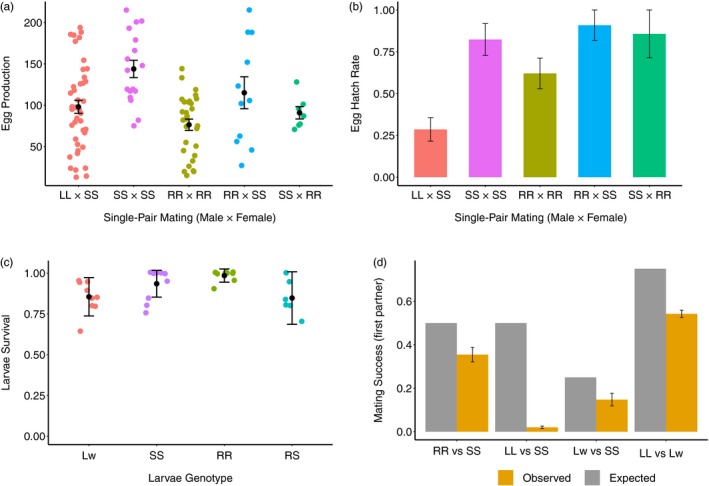
Fitness costs associated with self‐limiting transgenes and *Bt* resistance alleles in *Plutella xylostella* in this study. (a) Egg production of successfully mated (>10 eggs) single pairs of LL male × SS female, SS male × SS female, RR male × RR female, RR male × SS female and SS male × RR female. Black circles and error bars represent the mean egg production (±*SE*). (b) Egg hatch rate (±*SE*; >10 larvae emerged) of successfully mated single pairs. (c) Larvae survival of larvae genotype Lw, SS, RR and RS. Black circles and error bars represent the mean larvae survival (±*SE*) for respective genotypes. (d) Mean mating success (±*SE*) of RR males (vs. SS males—in competition with SS males), LL males (vs. SS males), Lw males (vs. SS males) and LL males (vs. Lw males). Yellow bars and error bars represent the mean observed mating success (±*SE*), while grey bars represent the expected mating success. All males denoted as LL and Lw were homozygous‐susceptible (SS), and all SS and RR individuals were nontransgenic (ww)

We assessed the fitness cost of the self‐limiting gene and the resistance allele in single‐pair crosses and mate competition experiments. In the single‐pair mating experiment, successful mating was defined as mating that resulted in more than 10 eggs. Only eggs from successful matings were counted and used to estimate fecundity and hatch rate as mating efficiency was assessed in competition experiments. The genotype of mating partners had a strong impact on fecundity (Figure 4a, *F*_4,101_ = 5.69, *p* < .001), with highest fecundity in VB‐S individuals (SS × SS) and lowest fecundity in VB‐R individuals (RR × RR; Figure 4a). Single pairs of LL male × SS female had an intermediate level of egg production. From the counted eggs, we estimated egg hatch rate as the percentage of successfully mated single pairs that had eggs developed into more than 10 neonate larvae. As the self‐limiting construct eliminates female progeny at larval stage (Jin et al., 2013), we would expect that the rate of egg hatch would be similar to that of wild‐type insects. However, the egg hatch rate in LL male x SS female mating was significantly lower than wild‐type pairs (Figure 4b, χ^2^ = 6.88, *df* = 1, *p* = .01). Single pairs of LL male × SS female had a significantly lower egg hatch rate than all other mating genotypes (Figure 4b, *F*_4,101_ = 6.68, *p* <<< .001). Larval survival was defined as the proportion of neonate larvae that developed into pupae in 10 days. There was no significant difference in larval survival between genotypes (Figure 4c, χ^2^ = 1.303, *df* = 3, *p* = .73).

In the mating competition experiment, if RR males and LL males were equally as competitive as SS males for mating with SS females, we would expect half of the offspring to be RS individuals (scored by PCR) or Lw individuals (scored by red fluorescence), respectively. Contradicting our null hypothesis, both RR males (Figure 4d, χ^2^ = 16.58, *df* = 1, *p* < .001) and LL males (Figure 4d, χ^2^ = 591.14, *df* = 1, *p* <<< .001) had lower mating success than expected. Similarly, under random mating, with competition between heterozygous Lw males and SS males, a quarter of the male progeny should be fluorescent Lw individuals. Again, contradicting our null hypothesis, Lw males produced fewer progeny than expected (Figure 4d, χ^2^ = 7.79, *df* = 1, *p* = .0053), indicating that the fitness costs associated with the transgene are incompletely dominant. Finally, for LL males in competition with Lw males (mating with SS females), significantly less than 75% of the male progeny were fluorescent, indicating that homozygous LL males had lower mating success than their heterozygous Lw counterparts (χ^2^ = 209.21, *df* = 1, *p* <<< .001). In a population of mixed genotypes, the hierarchy of mating success of males would be wild type (ww) > Lw > LL and SS > RR (Figure 4d).

## 4 DISCUSSION

Here, we have investigated the role of transgenic insect releases in mitigating levels of resistance and suppressing population growth in DBM. We found good support for population suppression and resistance reduction with the combined use of the high‐dose/refuge strategy and self‐limiting transgenic DBM (Alphey et al., 2007, 2009). The most straightforward evidence was that the transgenic DBM males were able to suppress both population size and resistance development (Figure 2).

Here, we found effects on the evolution of resistance in this even though refuge size (10%) and the initial resistance allele frequency (15%) in this work were substantially smaller and higher, respectively, than are typical in the field (Tabashnik et al., 2008). Moreover, in comparison with conventional sterile insect technique programmes, which could release typically 10, or even up to 50 sterile males to one wild‐type male (Dyck, Hendrichs, & Robinson, 2005; Lees, Gilles, Hendrichs, Vreysen, & Bourtzis, 2015), the release ratio of 6:1 of the self‐limiting DBM is relatively modest.

Given the success of the self‐limiting DBM, several factors could potentially limit the effect of the transgenic males. Our data demonstrated that the release of the self‐limiting males had a greater impact on resistance frequency at smaller refuge size (Figure 1a). Potentially, the strong effect of refuge size on slowing the evolution of resistance can mask the effect of the transgenic males at a low release ratio, rendering the effect of release undetectable (Figure 1b). Put simply, if the refuge strategy is working well to suppress the evolution of resistance, then there are limited gains to be had from the additional release of transgenic males. Notably, while resistance frequencies are low, the insect population growth rate will be determined by refuge size (Tellez‐Rodriguez et al., 2014), so larger refugia can also mask effects on population suppression. Simulation modelling indicated that very effective resistance (RR individuals have survival rates that approach 100% on Cry1Ac diet in these experiments) and low fitness costs of resistance could mask the effects of self‐limiting transgenes at low release ratios (Supporting Information: modelling). Over multiple discrete generations, other forms of fitness costs such as delayed developmental time of the self‐limiting males could also limit efficacy, while continuous, overlapping insect populations might be able to accommodate the other potential fitness costs (Supporting Information: modelling).

Release of insects carrying female‐specific self‐limiting transgenes should allow the build‐up of transgenic alleles over multiple generations, and we found clear evidence of population suppression and resistance reduction in continuous, overlapping DBM populations. Experiments initiated with 15% initial R allele frequency produced consistent results in terms of population size and proportion of resistance alleles. In contrast, with initial resistance alleles at 4%, transgenic males reduced population size and survival on toxin diet in experiments (Figure 3a,b), but we observed no difference in resistance development between the release‐treatment and no‐release populations (Figure 3c). The combined use of refugia and transgenic release meant that there was minimal survival on Cry1Ac diet, and therefore minimal selection for resistance. However, cages experienced population bottlenecks, particularly in replicates treated with transgenic males. Population bottlenecks can lead to increased variability in allele frequencies via drift (Hartl & Clark, 1997). The bottleneck effect could explain the marked variation in resistance allele frequencies in the release‐treatment populations.

Overall, transgenic males release could slow the evolution of resistance in repeated experiments, albeit at a reduced rate than that predicted by theory (Alphey et al., 2007, 2009). According to the published models, a lower release ratio was associated with effective resistance management consequences when refugia are larger and R allele frequencies lower than in our experiments (Alphey et al., 2007, 2009). At 10% refuge size and 10% initial R allele frequency, the release ratio of 1:1 was capable of slowing resistance development (Alphey et al., 2007, 2009), but in our experiment we released five or six transgenic males to every wild‐type male to achieve similar effects. As a consequence, we examined whether unforeseen impacts of transgenes and resistance alleles on life history traits (not reflected in the models) might explain this discrepancy.

Homozygous‐resistant individuals had reduced fitness as a result of lower fecundity and fertility (Figure 4a,b). Homozygous‐resistant individual males also had reduced mating success with susceptible females (Figure 4d). In the mating competition experiment, the males and females were introduced into the mating cages as emerged adults; it is unlikely that the mating success of tested males was correlated with different development times and population structure (Liu et al., 2001). Male mating success in the studied system may be associated with reduced number of matings, as seen in previous experiments with the NOQA, the DBM line that provided the resistance alleles for our population (Groeters, Tabashnik, Finson, & Johnson, 1993). Reduced fitness for RR individuals improves resistance management generally (Carrière & Tabashnik, 2001), but nonrandom mating could obstruct the effectiveness of the high‐dose/refuge strategy (Gould, 1998; Tabashnik et al., 2009).

Our results showed that the self‐limiting males were less competitive than wild‐type males in terms of accessing wild‐type females (Figure 4d) and that these matings resulted in fewer hatched eggs relative to wild‐type counterparts (Figure 4b). The fitness of the transgenic males was greatly reduced, as very few progeny were produced and survived from the mating. In addition, heterozygous transgenic males, which are responsible for introgressing susceptible alleles into the population at large, showed incompletely dominant fitness costs associated with transgenes (Figure 4d). If transgenes reduce the fitness of heterozygous males, then the potential for introgression of pesticide susceptibility alleles will be limited and the genetic consequences of release will approximate that of “bisex‐lethal” strains rather than female‐specific lethal. The process of building up the L allele frequency through releases over multiple generations, and its consequences for population suppression, will be attenuated by high dominant fitness costs. Critically, the efficacy of self‐limiting transgenic insects as tools in resistance management (above and beyond their use in population suppression) will be partly dependent on the dominance and degree of fitness costs associated with transgenes.

These fitness costs are higher than previously described for this DBM strain, potentially a result of variation in rearing conditions between laboratories (Harvey‐Samuel, Ant, Gong, Morrison, & Alphey, 2014; Jin et al., 2013), or because of the effects of differences in genetic background of nontransgenic insects arising from outcrossing wild‐type lines with NO‐QAGE (Raymond et al., 2011). Note that transfer of *P. xylostella* OX4139 line L from Oxitec to laboratories at Cornell also resulted in increased fitness costs (A. Walker, unpubl. dat.), which were partly ameliorated by reducing the temperature under which larvae are reared. We also saw weaker population suppression of insects than in earlier experiments with self‐limiting *P. xylostella* on broccoli plants (*Brassica oleracea*) expressing Cry1Ac (Harvey‐Samuel et al., 2015). In contrast to that study, we introduced toxin‐free refugia, which can substantially increase the reproductive potential of a population when resistance frequencies are low. In addition, in this study experiments used artificial diet, which imposes minimal mortality on early instars, whereas *B. oleracea* can cause substantial mortality on neonates, rising to 70% for genotypes resistant to Cry toxins (Raymond et al., 2011). Both these factors would facilitate population suppression on broccoli plants.

It is difficult to assess how relevant experiments conducted in caged insect population are for real‐world resistance dynamics. We hope that mate competition experiments in the laboratory capture sufficient naturalistic behaviour to be able to reflect what might happen in the field. The effects of relatively small population sizes can clearly impose some limitations and create additional variability when gene frequencies are low. Nevertheless, we have constructed experimental conditions that pose a very challenging scenario for resistance management. Frequencies of resistance alleles were high, refugia sizes were small and the release ratios low (Dyck et al., 2005). For diamondback moth on artificial diet, the fitness costs of resistance were relatively modest and resistant insects had survival rates of up to 100% on diet containing very high levels of Cry toxins, a situation that does not occur in the field, even in insect species prone to evolve resistance to *Bt* toxins readily (Tellez‐Rodriguez et al., 2014). In addition, while fitness costs of transgenes resulted in reduced mate competitiveness, in comparison to previous experiments with P. xylostella (index of competitive ability estimated at 0.09, where equal fitness =1), (estimated at 0.09 in this study, where equal fitness with wild type = 1)*,* competitive ability was higher than that observed for *Aedes aegypti* (0.008–0.31; Carvalho et al., 2015), suggesting that our experiments are not unrealistic in this regard. Thus, even under relatively stringent experimental conditions, our results suggest that the self‐limiting DBM is a promising strategy, compatible with the high‐dose/refuge strategy.
