# Supplementary material for: Combining the high‐dose/refuge strategy and self‐limiting transgenic insects in resistance management—A test in experimental mesocosms
Source: Evol Appl. 2018 Jan 18;11(5):727–38. doi: 10.1111/eva.12573 (PMC5979637; doi:10.1111/eva.12573)
Supplement: Supplementary file 1 [file EVA-11-727-s001.docx]

**Supplementary Information for “Combining the high-dose/refuge strategy and self-limiting transgenic insects in resistance management- a test in experimental mesocosms”.** Zhou et al. Evolutionary Applications.

**Toxin bioassays**

The activity of the Cry1Ac toxin was assessed in search for a recessive dose for resistance. Diet was prepared with toxin doses of 0.262 μg ml^–1^ and 0.524 μg ml^–1^ in 55 x 14 mm Petri dish, measuring 10 ml per Petri dish. Ten 3^rd^ instar *P. xylostella* larvae were placed onto the toxin diet and larval survival was measured after 5 days. *P. xylostella* larvae were also tested on diet with 0.85% NaCl solution as controls. For each toxin dose and three different *P. xylostella* populations, sixty larvae were tested. The *P. xylostella* populations used were the Cry1Ac resistant population NO-QAGE, the susceptible population Vera Beach (VB) and first generation cross of NO-QAGE and VB (F1).

**Table S1** Final larval survival after feeding on toxin and control diet for 5 days

| Diet | NO-QAGE | VB | F1 cross |
| --- | --- | --- | --- |
| 0.262 μg ml^–1^ Cry1Ac | 97.92% | 1.67% | 0% |
| 0.524 μg ml^–1^ Cry1Ac | 97.92% | 0% | 0% |
| 0.85% NaCl | 100% | 94.74% | 100% |

The Cry1Ac toxin concentration of 0.524 μg ml^–1^ caused 100% larval death of VB and F1 populations. On the other hand, the survival rate of NO-QAGE population was 97.92%. The Cry1Ac toxin dose of 0.524 μg ml^–1^ was sufficient to cause near-recessive resistance.

**Modelling: underlying population genetics model**

This work uses and develops a previously published deterministic, discrete-generation population genetic model of a closed, homogeneous population with random mating and a 1:1 sex ratio ([Alphey et al., 2009](#_ENREF_1); [Alphey et al., 2007](#_ENREF_2)). “Wild” populations are initiated with non-transgenic pupae (absence of the transgene is labelled as genotype ww) from a large laboratory stock population with known resistant R allele frequency (and assumed to be in Hardy-Weinberg equilibrium at the S/R locus). “No release” (NR) simulated treatments consist only of that wild population. “Released” males homozygous for the OX4319 genetic construct with dominant female-specific lethality (“L allele” at L/w locus) and homozygous susceptible to Bt toxin (SSLL genotype) are added for the simulated treatments with release of genetically engineered (“GE”) males. Adults emerge. After random mating, a fixed fraction of all progeny are allocated to Bt (toxin diet) and the remainder to refuge (toxin-free diet), preserving the genotype frequencies in both regimes. Larvae, the life stage that is susceptible to Bt toxins, spend all their development time either on toxin diet or on refuge diet. Fitness costs and advantages of resistance (R allele) take effect during the larval stage, by applying relative fitness fractions (survival relative to SSww) in each dietary regime, followed by lethality to females caused by the engineered OX4319L construct (LL and Lw females do not survive, males are unaffected). Dispersal occurs prior to mating, and is enacted in the experiments by pooling survivors from toxin and refuge diet within each replicate. Those progeny are then reared on non-toxin diet to late II or early III instar larvae, with refuge fitness costs being applied in the model, prior to being sampled for the bioassay.

**One-generation experiments: deterministic model**

| Default parameter values:  [experimental, planned]  initial R allele frequency *p*_0_ 0.15  refuge 5%  release 2 SSLL males for every non-transgenic male (equal sexes assumed)  [relative fitness values, data/estimates]  in refuge SS 1, SR 1, RR 0.95, i.e. fitness costs of resistance are recessive  on Bt SS 0, SR 0, RR 0.9, i.e. resistance is incomplete and recessive, toxin is high-dose  Lw or LL females 0 (lethal), males 1 (no cost), i.e. perfect genetic construct |
| --- |

The existing deterministic model was used to explore issues relevant to experimental design.

Inaccuracy in allocating eggs to toxin or refuge

If the oviposition substrate is not cut precisely, or the eggs laid are not evenly spread across the substrate, the actual allocation of eggs between toxin and refuge diets might not be equal to the intended experimental proportions. We used the deterministic model to assess the effect such inaccuracy might have on the outcomes, with intended refuge at either 5% or 20%. Table S1 shows the simulated R allele frequency in the progeny reared through larval stages, after the effects of selection (toxin diet, fitness costs of resistance) and genetic lethality of females, and prior to being sampled for the resistance bioassay. Inaccurate allocation to refuge can have a material effect on R allele frequencies, particularly at smaller refuge sizes. However, it does not make much difference to the relative reduction in R allele frequency caused by the GE release treatment.

**Table S2** Refuge size not as intended

| R frequency among late larvae prior to bioassay (to 3 d.p.) | GE release | No release | Relatively  GE/NR  (to 2 d.p.) |
| --- | --- | --- | --- |
| Default except refuge 4% | 0.335 | 0.422 | 0.79 |
| Default parameters (refuge 5%) | 0.302 | 0.381 | 0.79 |
| Default except refuge 6% | 0.277 | 0.350 | 0.79 |
| Default except refuge 18% | 0.177 | 0.219 | 0.81 |
| Default except refuge 20% | 0.172 | 0.211 | 0.82 |
| Default except refuge 22% | 0.167 | 0.204 | 0.82 |

RR frequency measured by the bioassay

The bioassay measures the resistant phenotype frequency (RR frequency, as toxin doses are high enough to render resistance functionally recessive). This is a valid measure to demonstrate any resistance dilution effect of GE releases. However, it cannot be used to compute the R allele frequency directly (without knowledge of the selection coefficients, i.e. the relative fitnesses), because the RR frequency in late II / early III instar larvae is not equal to the square of the R allele frequency.

Alleles are in Hardy-Weinberg equilibrium immediately following reproduction, i.e. in zygotes, eggs, and (with our assumptions of identical egg hatch rates) neonate larvae. After that, selection reduces survival. Following the selection, survivors are mated and progeny reared on toxin-free diet to late II / early III instar larvae in preparation for the bioassay. In the model, RR (and potentially SR) types suffer mortality due to fitness costs of resistance, and any transgenic females (progeny of male offspring of the released SSLL GE males) die from the engineered lethality.

The deterministic model showed that, as an estimate, the RR frequency is not expected to be materially different from the square of the R allele frequency. Table S2 gives example results.

**Table S3** Resistant phenotype and R allele frequencies

| among II/III larvae prior to bioassay (to 3 d.p.) | GE release | | No release | |
| --- | --- | --- | --- | --- |
|  | R frequency squared | RR frequency | R frequency squared | RR frequency |
| Default parameters | 0.091 | 0.082 | 0.145 | 0.142 |
| Default except refuge 20% | 0.029 | 0.027 | 0.045 | 0.043 |
| Default except 0.5 RR fitness on non-toxin diet | 0.055 | 0.050 | 0.085 | 0.088 |

Bioassays with 90 larvae to detect resistance dilution

Pupae surviving experimental treatments are pooled (within the replicate), and allowed to emerge as adults and mate. Eggs are collected and reared on non-toxin diet to II/III instar, of which 90 (in pilot experiments) are transferred to toxin diet and the numbers surviving to pupate are counted, giving the frequency of the resistant phenotype (assumed RR). The number of survivors (out of 90) must be an integer, and this coarse-grained set of possible results, together with stochastic variation in survival, might mask any resistance dilution effect. For example, in Table S3, with 20% refuge and otherwise default parameter values, 2 or 3 individuals are predicted to survive from the GE treatment (2.43) and 3 or 4 are predicted to survive from the no release treatment (3.87). Consequently, the number of individuals to use in bioassays was reconsidered and this potential experimental bottleneck was identified for stochastic modelling.

**Table S4** Potential inability to discriminate outcomes from bioassay samples

|  | GE release | | No release | |
| --- | --- | --- | --- | --- |
| (to 3 d.p.) | RR frequency among II/III larvae prior to bioassay | RR survivors (of 90) of bioassay | RR frequency among II/III larvae prior to bioassay | RR survivors of bioassay |
| Default parameters | 0.082 | 7.38 | 0.142 | 12.78 |
| Default except refuge 20% | 0.027 | 2.43 | 0.043 | 3.87 |
| Default except 0.5 RR fitness on non-toxin diet | 0.050 | 4.5 | 0.088 | 7.92 |
| Default except GE 1:1 | 0.102 | 9.18 | 0.142 | 12.78 |

More widespread resistance initially

Parameters default values (as above) except:

initial R allele frequency *p*_0_ 0.15 (default) or 0.44 (alternative experiment)

release *3* SSLL males for every non-transgenic male (equal sexes assumed),

for comparison with later stochastic models below

A higher initial frequency of resistant alleles in the population (*p*_0_) would lead to greater numbers of individuals surviving the toxin diet. However, more widespread resistance was previously predicted to be harder to reverse with dilution using susceptible GE males carrying female-lethal constructs, for example needing a higher combination of GE release and refuge ([Alphey et al., 2009](#_ENREF_1)).

With a 3:1 release ratio, and all other assumptions unchanged, performing the same experiment with initial allele frequency 0.44 instead of 0.15 would roughly halve the size of the effect (lower RR genotype frequency due to GE release) to be detected.  See Table S4. Instead of looking for evidence of a 50% reduction, we would be looking for a 26% reduction in proportion surviving bioassays. The default value of *p*_0_ 0.15 is appropriate for the experiments.

**Table S5** Initial R allele frequency impact on outcomes

|  | *p*_0_=0.15 | | *p*_0_=0.44 | |
| --- | --- | --- | --- | --- |
| Release engineered males or none | GE | NR | GE | NR |
| R allele frequency in late larvae | 0.287 | 0.381 | 0.751 | 0.865 |
| RR frequency (bioassay survivors) | 0.071 | 0.142 | 0.550 | 0.747 |
| Predicted effect of GE treatment (compared to NR) on frequency of RR types | Reduce by 50% | | Reduce by 26% | |

**One-generation experiments: stochastic modelling**

Default parameter values: as for deterministic model (above)

Notation, diamondback moth populations:

VB: Vero Beach strain, susceptible, assumed to be SSww.

NO-QAR: “resistant strain”, includes resistant alleles, SSww, SRww and RRww.

Fx: created from individual crosses VB x NO-QAR, resistant allele frequency 0.35 estimated by PCR of a sample.

Fz: created from individual crosses VB x Fx, aiming for 0.15 resistant allele frequency.

The source populations (VB, Fx, Fz) comprise thousands of moths and can be regarded as “very large” for modelling purposes. We hypothesised that stochastic effects in drawing the initial small populations to begin the experiments could help explain lack of observed effect of GE releases in the first pilot experiments.

In the first experiments, 200 non-transgenic pupae were inserted into each mating cage, comprising 96 from the VB population and 104 from the Fx population. For the GE treatments, 100 or 200 GE male pupae were also introduced into the cages. For the next experiments, an Fz population was created, with (approximately) the desired R allele frequency, from which to select all pupae representing the natural population in the experiments.

Variability in the small mating cage populations

A stochastic spreadsheet model was developed, based on the deterministic model, and differs as follows:

- Instead of assuming equal numbers of males and females in the initial population, each pupa has a 0.5 chance of being female – the number of females among *n* pupae to be drawn from each source population is generated by Binomial distribution B(*n*,0.5).
- Pupae drawn from Fx or Fz populations (ww, non-transgenic) are assigned genotypes SS, SR or RR, by assigning each allele of the S/R pair independently. For each sex separately,
  - the number of pupae with first allele (maternally donated, say) being S is generated by Binomial distribution B(*n*,*q*), where *n* is the number drawn of that sex and *q* (=1-*p*) is the S allele frequency in the source population,
  - then within each of those subsets (for each sex, 1^st^ allele S or R), the number with second allele S is generated by B(*n*,*q*) where *n* is the number in that subset and *q* is the S allele frequency in the source population.
- The number of released SSLL males is the specified multiple of the expected number of males, assuming that half the non-transgenic individuals are male, which may not actually be true.

Together, these calculations give the genotype frequencies (by S/R locus, L/w locus and sex) of the pupae in at the start of the experiment. Thereafter, we assume that gametes are produced in proportion to their frequencies, allocation of eggs to refuge or toxin diet is accurate, and the spreadsheet calculations are as before.

For each of the no release (NR) and GE release treatment, 200 stochastic realizations were generated for the first pilot experiments (“VB+Fx”, drawn from two source populations as described above) and, separately, for the follow-on experiments (drawn from “Fz” source population).

Results show that his source of variability alone could be sufficient to mask any effect of GE release treatment on dilution of the R allele. The variation is similar in magnitude whether drawing pupae from a mixture of VB and Fx populations, or from the FZ population. Table S5 shows summary results with default parameters.

**Table S6** Stochastic variability in drawing pupae for initial experimental set-up

| R allele frequency (3 d.p.) | | Deterministic | Stochastic (200 realizations) | | |
| --- | --- | --- | --- | --- | --- |
|  |  |  | Mean±S.E. | Maximum | Minimum |
| VB+Fx | GE release | 0.345 | 0.345±0.042 | 0.465 | 0.219 |
|  | No release | 0.433 | 0.422±0.044 | 0.558 | 0.286 |
| Fz | GE release | 0.345 | 0.339±0.047 | 0.477 | 0.220 |
|  | No release | 0.433 | 0.434±0.053 | 0.559 | 0.300 |

The mean results over 200 stochastic realizations have not quite converged to the deterministic results in the Fz case, but are fairly close. The Mean±S.E. ranges of GE release and no release overlap, and there is potentially wide variability about the mean. As noted above, there will also be inaccuracy in estimating the outcome R allele frequencies, because the RR frequency measured (bioassay survivors, post-selection) is not equal to the square of the R allele frequency.

A key outcome is the simulated reduction in R allele frequency (due to GE treatment, compared with NR treatment), which is expressed in Table S6 as a percentage $\left( 1-\frac{p_{GE}}{p_{NR}} \right)\times100\%$. The expected reduction due to the GE release treatment is 20%, but the experiment can give a wide range of values. The Mean±S.E. ranges do not overlap zero, but might be close enough that the experimental measurements could be unable to discriminate, and some realizations actually generated negatives i.e. in some instances the GE treatment showed higher R allele frequency than observed with no release.

**Table S7** Dilution of resistance by GE release treatment

| Reduction in R allele frequency with GE release compared to no release (%, 0 d.p.) | Deterministic | Stochastic (200 realizations) | | |
| --- | --- | --- | --- | --- |
|  |  | Mean±S.E. | Maximum | Minimum |
| VB+Fx | 20% | 17±14% | 53% | 34% increase |
| Fz |  | 21±15% | 56% | 23% increase |

Mating cage population size effect on discriminatory power of experiment

Parameters default values (as above) except:

release *3* SSLL males for every non-transgenic male (equal sexes assumed)

The hypothesis being tested is one-sided: the R allele frequency, and hence the RR genotype frequency, in bioassayed larvae is lower with GE treatment than with NR treatment. 250 stochastic realizations were simulated for each of the NR (no release) and GE treatments for any given mating cage size. If the 5^th^ percentile of NR results RR frequency is lower than the 95^th^ percentile of GE results RR frequency, i.e. the one-sided 95% ranges do not overlap, this experimental set up should be able to distinguish between GE and NR treatments (on the assumption that the initial population of the mating cages is the only source of significant variability). In these circumstances it is very unlikely that a lower RR frequency in the GE treatment than in the NR treatment is due to random chance.

In these simulations, either 400 or 1000 non-transgenic pupae were inserted into each simulated mating cage, drawn from a population with 0.15 R allele frequency. For the GE treatment, 600 or 1500 GE male pupae, respectively, were also introduced into the cage. This is a “release ratio” of 3:1 GE to non-transgenic males, assuming equal sexes (the original default was 2:1 – this slightly higher ratio would give a greater effect that should be easier to detect). Summary results are shown in Table S7. The expected reduction in R allele frequency with GE compared to NR, $1-\frac{p_{GE}}{p_{NR}}$ expressed as a percentage, is 24.7%. Some realizations generated negatives i.e. an increase in R allele frequency observed with the GE treatment compared to no release. The mating cage size of 400 is sufficient that the 5^th^ percentile of NR results RR frequency is lower than the 95^th^ percentile of GE results RR frequency.

**Table S8** Size of mating cage initial population

| (to 3 d.p.) | | | Deterministic result | Stochastic results (250 realizations) | | |
| --- | --- | --- | --- | --- | --- | --- |
|  |  |  |  | Mean | S.E. | 95^th^ percentile (GE), 5^th^ percentile (NR) |
| Mating cage 400  (+ 600 GE males) | GE | R allele frequency in late larvae (*p*) | 0.287 | 0.283 | 0.030 |  |
|  |  | *p*^2^ | 0.082 | 0.081 | 0.017 |  |
|  |  | RR (bioassay survivor frequency) | 0.071 | 0.070 | 0.015 | 0.097 |
|  | NR | R allele frequency in late larvae (*p*) | 0.381 | 0.379 | 0.038 |  |
|  |  | *p*^2^ | 0.145 | 0.145 | 0.029 |  |
|  |  | RR (bioassay survivor frequency) | 0.142 | 0.142 | 0.029 | 0.099 |
|  | Reduction in R frequency  $\left( 1-\frac{p_{GE}}{p_{NR}} \right)\times100\%$ | | 24.7% | 24.7% | 10.9% |  |
| Mating cage 1000  (+ 1500 GE males) | GE | R allele frequency in late larvae (*p*) | 0.287 | 0.287 | 0.030 |  |
|  |  | *p*^2^ | 0.082 | 0.083 | 0.017 |  |
|  |  | RR (bioassay survivor frequency) | 0.071 | 0.072 | 0.015 | 0.098 |
|  | NR | R allele frequency in late larvae (*p*) | 0.381 | 0.382 | 0.037 |  |
|  |  | *p*^2^ | 0.145 | 0.147 | 0.029 |  |
|  |  | RR (bioassay survivor frequency) | 0.142 | 0.144 | 0.028 | 0.100 |
|  | Reduction in R frequency  $\left( 1-\frac{p_{GE}}{p_{NR}} \right)\times100\%$ | | 24.7% | 24.0% | 11.2% |  |

In these simulations, the only stochasticity was drawing the pupae from the stock (Fz) population to initiate the mating cages. As discussed above, the number of larvae subjected to the bioassay must also be sufficient to distinguish between RR survivors frequency expected values of 0.142 (NR) and 0.071 (GE), so these two experimental bottlenecks were next modelled together.

Number of larvae used for bioassay

Parameters default values (as above for one-generation models generally) except:

release *3* SSLL males for every non-transgenic male (equal sexes assumed)

The stochastic model was extended to include random drawing of larvae to use in the resistance bioassay, as well as the random initiation of mating cages already modelled. The purpose was to explore how large a bioassay sample should be sufficient to distinguish between treatments.

Again, 400 non-transgenic pupae were inserted into each simulated mating cage. For the GE treatment, 600 or 1500 GE male pupae, respectively, were also introduced into the cage (“release ratio” of 3 RIDL : 1 non-transgenic males, assuming equal sexes).

The stochasticity in this version of the model is:

- As before, each (non-transgenic) pupa inserted in the mating cage has a 0.5 chance of being female;
- As before, pupae drawn from the source population are assigned genotypes SS, SR or RR, by Binomial distributions;
- A new feature - each early larva drawn from progeny of the test cages is assigned a genotype (e.g. male SSLw, female SRww, …) by assigning sex and each allele independently, using repeated Binomial distributions with probabilities equal to the simulated genotype frequencies among the eggs laid by first generation adults.

250 stochastic realizations were simulated for each of the NR and GE treatments, for mating cage size 400 (+600 GE males in the GE treatments) and bioassay sample size 90, 180, 300 or 450.

Summary results are in Table S8. Mean results are similar to those in Table S7, the results with stochasticity only in mating cage initiation. However, there is greater variability in outcomes. For example, the previous model had standard error (S.E.) of RR bioassay survivor frequency 0.015 GE and 0.029 NR, while with 90 larvae drawn stochastically for bioassay those S.E.s were 0.030 GE and 0.043 NR. None of these bioassay sample sizes modelled is sufficient that the 5^th^ percentile of NR results RR frequency is lower than the 95^th^ percentile of GE results RR frequency, i.e. the one-sided 95% ranges overlap, and this experimental set up may not be able to distinguish between GE and NR treatments. With larger bioassay sizes, the boundaries become close (with 450 bioassayed, the 95^th^ percentile GE is 0.102 and the 5^th^ percentile NR is 0.096), and might have passed a less strict test.

**Table S9** Combined stochasticity in initial mating cage set up and sampling for bioassay

| Mating cage 400 (+ 600 GE males)  (to 3 d.p.) | | | Deterministic result | Stochastic results (250 realizations) | | |
| --- | --- | --- | --- | --- | --- | --- |
|  |  |  |  | Mean | S.E. | 95^th^ percentile (GE) 5^th^ percentile (NR) |
| Bioassay 90 | GE | R allele frequency in late larvae (*p*) | 0.287 | 0.286 | 0.030 |  |
|  |  | *p*^2^ | 0.082 | 0.083 | 0.018 |  |
|  |  | RR (bioassay survivor frequency) | 0.071 | 0.069 | 0.030 | 0.128 |
|  | NR | R allele frequency in late larvae (*p*) | 0.381 | 0.379 | 0.036 |  |
|  |  | *p*^2^ | 0.145 | 0.145 | 0.027 |  |
|  |  | RR (bioassay survivor frequency) | 0.142 | 0.142 | 0.043 | 0.078 |
|  | Reduction in R frequency  $\left( 1-\frac{p_{GE}}{p_{NR}} \right)\times100\%$ | | 24.7% | 23.7% | 11.3% |  |
| Bioassay 180 | GE | R allele frequency in late larvae (*p*) | 0.287 | 0.285 | 0.028 |  |
|  |  | *p*^2^ | 0.082 | 0.082 | 0.016 |  |
|  |  | RR (bioassay survivor frequency) | 0.071 | 0.072 | 0.025 | 0.117 |
|  | NR | R allele frequency in late larvae (*p*) | 0.381 | 0.376 | 0.037 |  |
|  |  | *p*^2^ | 0.145 | 0.143 | 0.028 |  |
|  |  | RR (bioassay survivor frequency) | 0.142 | 0.138 | 0.035 | 0.089 |
|  | Reduction in R frequency  $\left( 1-\frac{p_{GE}}{p_{NR}} \right)\times100\%$ | | 24.7% | 23.6% | 10.3% |  |
| Bioassay 300 | GE | R allele frequency in late larvae (*p*) | 0.287 | 0.285 | 0.031 |  |
|  |  | *p*^2^ | 0.082 | 0.082 | 0.018 |  |
|  |  | RR (bioassay survivor frequency) | 0.071 | 0.072 | 0.022 | 0.110 |
|  | NR | R allele frequency in late larvae (*p*) | 0.381 | 0.379 | 0.036 |  |
|  |  | *p*^2^ | 0.145 | 0.145 | 0.027 |  |
|  |  | RR (bioassay survivor frequency) | 0.142 | 0.143 | 0.034 | 0.083 |
|  | Reduction in R frequency  $\left( 1-\frac{p_{GE}}{p_{NR}} \right)\times100\%$ | | 24.7% | 24.0% | 11.0% |  |
| Bioassay 450 | GE | R allele frequency in late larvae (*p*) | 0.287 | 0.284 | 0.029 |  |
|  |  | *p*^2^ | 0.082 | 0.081 | 0.016 |  |
|  |  | RR (bioassay survivor frequency) | 0.071 | 0.071 | 0.019 | 0.102 |
|  | NR | R allele frequency in late larvae (*p*) | 0.381 | 0.377 | 0.038 |  |
|  |  | *p*^2^ | 0.145 | 0.144 | 0.029 |  |
|  |  | RR (bioassay survivor frequency) | 0.142 | 0.142 | 0.034 | 0.096 |
|  | Reduction in R frequency  $\left( 1-\frac{p_{GE}}{p_{NR}} \right)\times100\%$ | | 24.7% | 23.9% | 11.2% |  |

These results suggested further modifications to experimental design.

**Three-generation experiments: deterministic modelling**

The frequency evolution of R alleles in one generation is limited. Running experiments for a small number of generations could potentially allow for a greater effect, which could be more readily detected with practical population and sample sizes. Three generations of the deterministic model were simulated to explore the potential advantage of extending experiments in time.

Parameters default values (as above for one-generation models generally) except:

initial R allele frequency *p*_0_ 0.51 (in line with a planned pilot experiment)

release 2 *or* *1* SSLL males for every non-transgenic male (equal sexes assumed)

Relative fitness values

RR various values as shown in Figure legends

Lw males 0.9 (i.e. 0.1 fitness cost),

(LL males never arise because the construct is 100% lethal to females)

The fitness traits of the resistant allele have a very material impact on whether or not the GE-treatment outcomes are close to those of the no-release treatment in the long run (see Figure S1 long term equilibria, and Figure S2 zoomed in on first four generations). If the resistance is quite effective (e.g. in (a) 4 out of every 10 RR insects survive on Bt toxin) and there are little or no fitness costs (in (a) 9 out of 10 RR insects survive on non-toxin diet), the effects of treatment may not be very large, particularly in the first few generations, and may be masked by the kind of stochastic variation described above. The fitness traits of the experimental resistant strain were unknown.

**Figure S1.** Graphs of R allele frequency over time


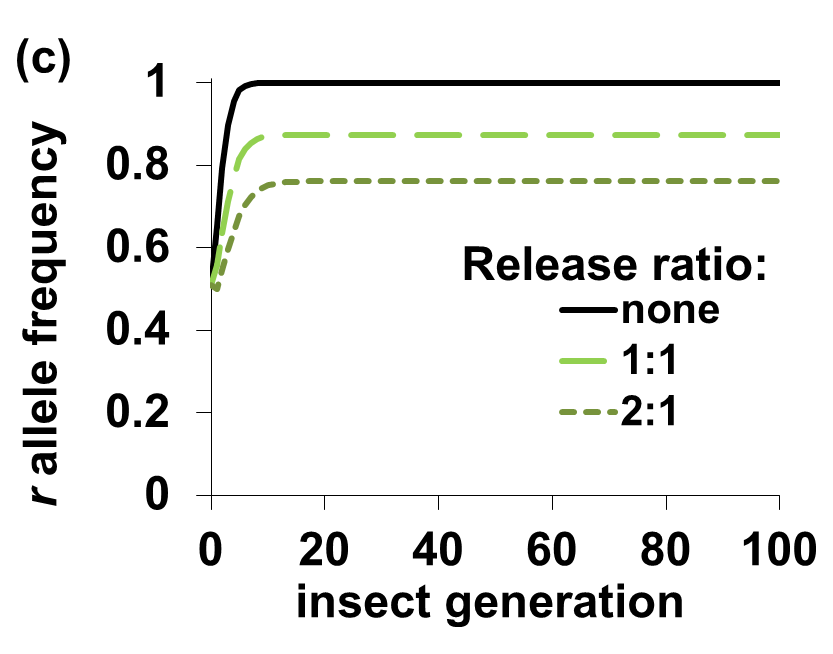

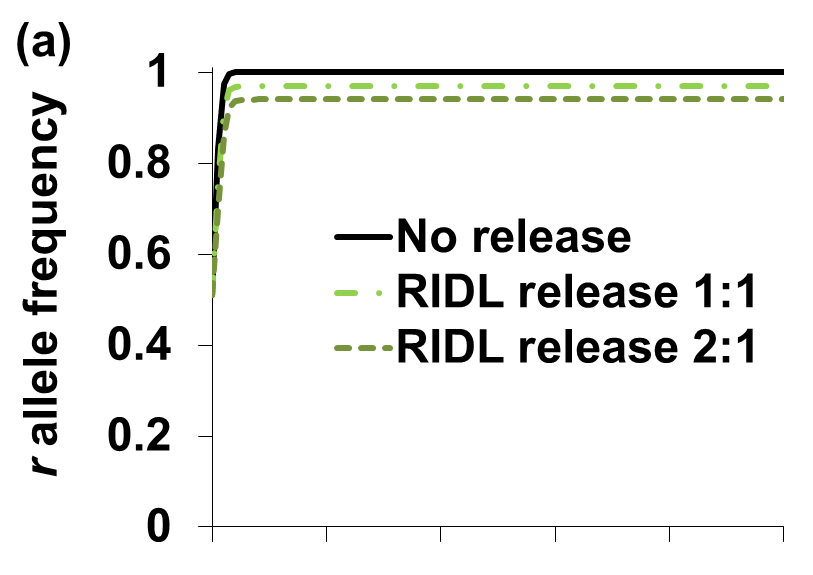

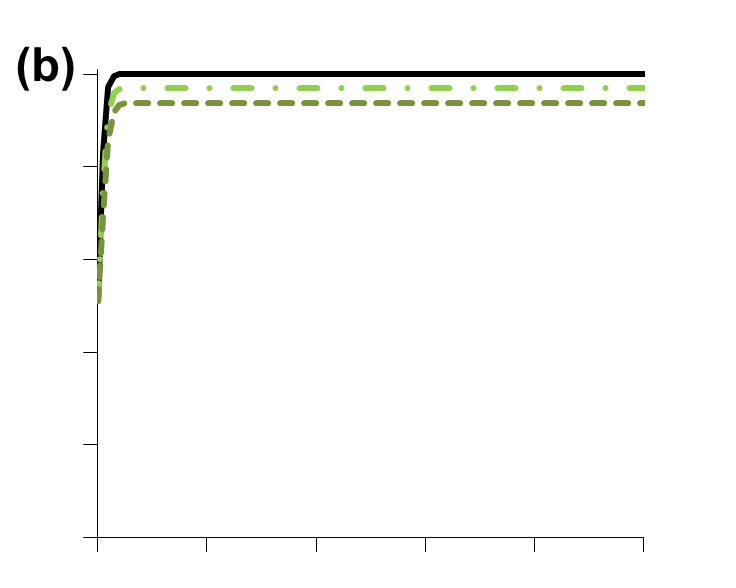

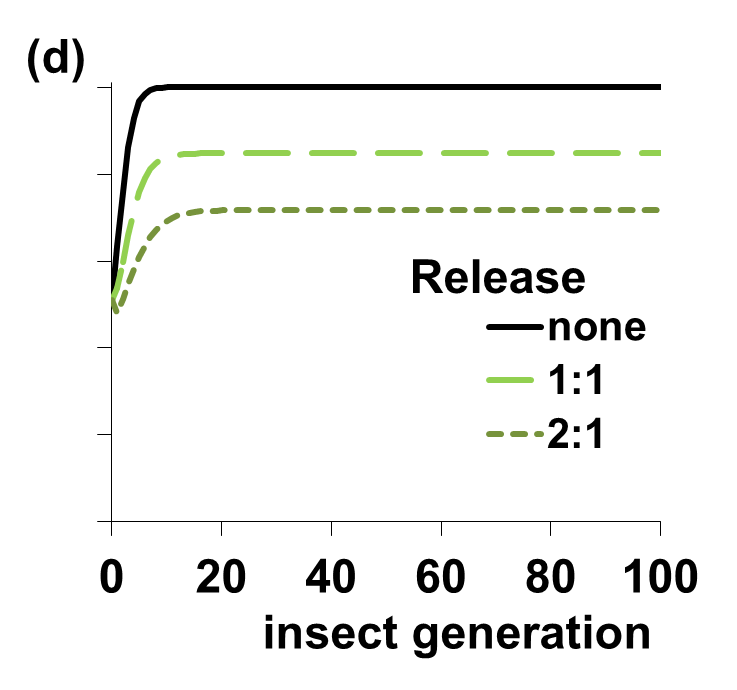


**R**

**R**

1. RR survival 0.4 on Bt, 0.9 in refuge (the strongest combination shown)
2. RR survival 0.4 on Bt, 0.7 in refuge (greater fitness costs than (a))
3. RR survival 0.1 on Bt, 0.7 in refuge (less effective resistance than (b))
4. RR survival 0.1 on Bt, 0.4 in refuge (the weakest shown, greater costs than (c))

**Figure S2** the same graphs as Figure S1 showing only generations 0 to 4


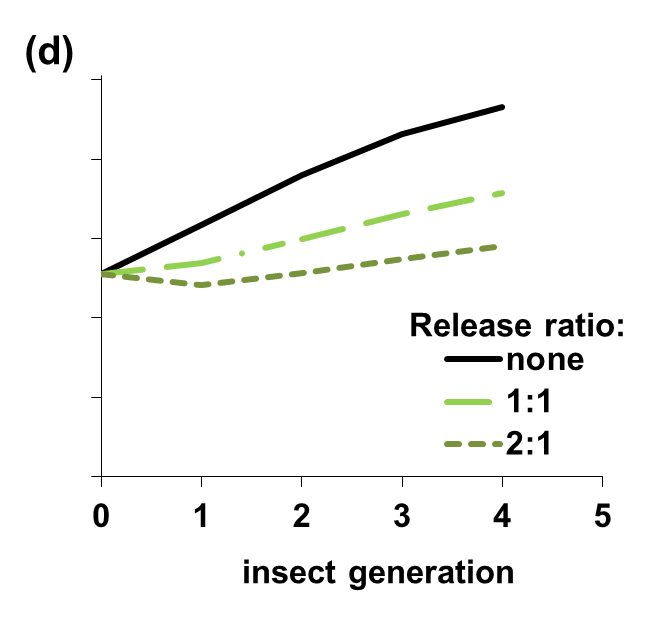

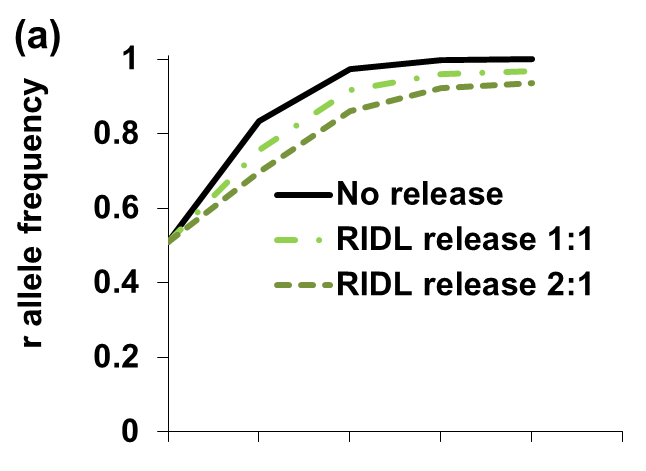


**R**


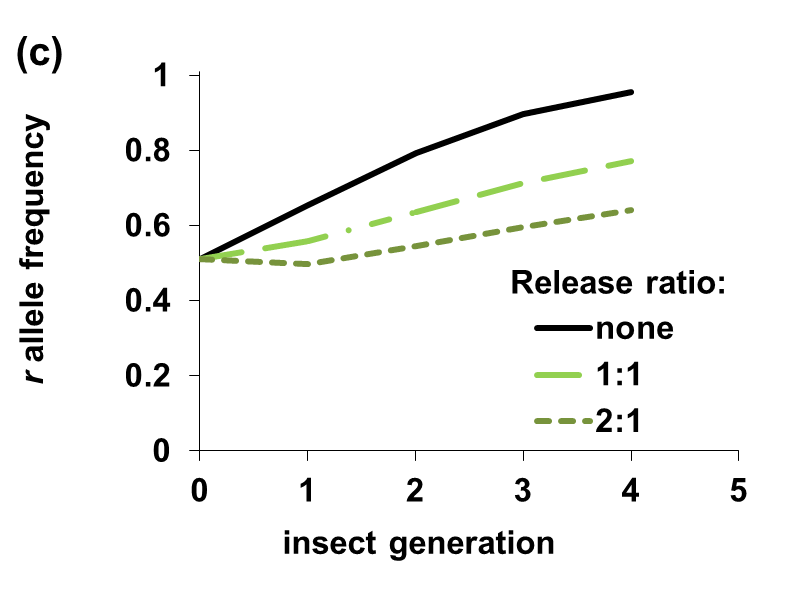


**R**


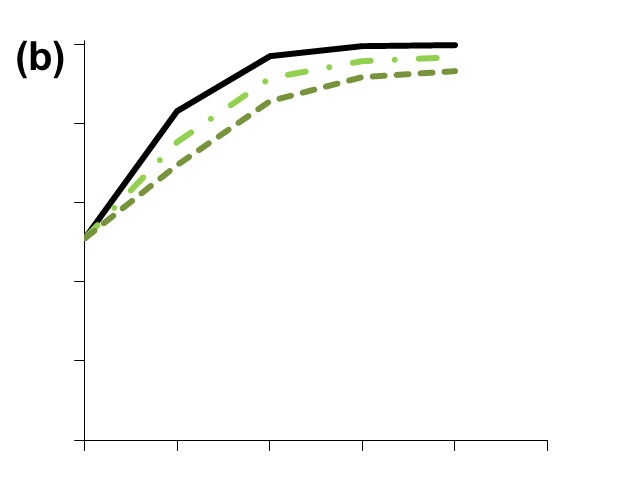


**(d)**

In these models, fitness costs of resistance and of the engineered construct were represented as reduced survival of larvae. In reality, larvae may suffer other forms of fitness penalties, such as delayed developmental time, which are not allowed for in discrete generation population genetic models. The final set of experiments was conducted with continuous, overlapping generations, rather than discrete generations, to accommodate these kinds of fitness costs.

**References**

Alphey, N., Bonsall, M.B. and Alphey, L. (2009) Combining Pest Control and Resistance Management: Synergy of Engineered Insects With Bt Crops. *Journal of Economic Entomology* 102, 717-732.

Alphey, N., Coleman, P.G., Donnelly, C.A. and Alphey, L. (2007) Managing insecticide resistance by mass release of engineered insects. *Journal of Economic Entomology* 100, 1642-1649.
